# Effects of κ-Carrageenan on Gel Properties and Microstructure of Unrinsed Nile Tilapia Surimi Gels During Heat-Induced Gelation

**DOI:** 10.3390/gels12080681

**Published:** 2026-08-02

**Authors:** Wanwen Chen, Xinyu Chang, Lanxian Yang, Jian Wu, Pao Xu, Hao Cheng, Haibo Wen

**Affiliations:** 1 Wuxi Fisheries College, Nanjing Agricultural University, Wuxi 214081, China; chenwanwen@ffrc.cn (W.C.);; 2 Key Laboratory of Integrated Rice-Fish Farming Ecology, Ministry of Agriculture and Rural Affairs, Freshwater Fisheries Research Center, Chinese Academy of Fishery Sciences, Wuxi 214081, China; 3 Sino-US Cooperative International Laboratory for Germplasm Conservation and Utilization of Freshwater Mollusks, Freshwater Fisheries Research Center, Chinese Academy of Fishery Sciences, Wuxi 214081, China; 4 College of Fisheries and Life Sciences, Dalian Ocean University, Dalian 116023, China; 5 State Key Laboratory of Food Science and Resources, School of Food Science and Technology, Jiangnan University, Wuxi 214122, China

**Keywords:** Nile tilapia, unrinsed surimi, κ-carrageenan, gel properties, intermolecular interactions, microstructure, water-holding capacity

## Abstract

Unrinsed surimi production has emerged as a sustainable alternative to conventional rinsed surimi due to its substantial water savings, reduced nutrient loss, and lower environmental footprint. However, poor gel strength and water-holding capacity remain major bottlenecks limiting its industrial application. This study investigated the effects of κ-carrageenan (κ-CG) on the rheological properties, textural characteristics, water distribution, intermolecular interactions, and microstructure of unrinsed surimi gels. The results showed that κ-CG enhanced gel properties in a concentration-dependent manner. At 0.75% κ-CG, gel strength increased to 47.0 g·cm (a 2.2-fold increase vs. control), water holding capacity (WHC) reached 97.9%, and cooking loss decreased to 4.0%. In contrast, at 1.0% κ-CG, gel strength and hardness further increased, but WHC declined slightly. Low-field nuclear magnetic resonance analysis revealed that κ-CG promoted the conversion of free water to immobilized water, with the relative peak area of T_22_ (immobilized water) increasing from 85.9% to 88.9% at 0.75% κ-CG. Protein solubility measurements indicated that hydrophobic interactions and disulfide bonds appeared to be the major contributors stabilizing the gel network. FTIR revealed enhanced hydrogen bonding via an O–H red shift and intensification with κ-CG, and amide I deconvolution showed that 0.75% κ-CG maximized β-sheet content while reducing α-helix, indicating ordered protein reorganization during heating. Quantitative SEM analysis further verified that 0.75% κ-CG produced the densest protein network with the maximum fractal dimension and minimum lacunarity, alongside reduced average pore area and porosity. These findings demonstrate that κ-CG at an appropriate level effectively improves the gel properties of unrinsed tilapia surimi by strengthening intermolecular interactions and optimizing water distribution, offering a practical approach for developing high-quality surimi products.

## 1. Introduction

Nile tilapia (*Oreochromis niloticus*) is one of the most economically important aquaculture species worldwide and a major contributor to global animal protein supply. Its annual production is expected to hit 7.3 million tons by 2030 [1]. Nile tilapia is characterized by rapid growth, high fecundity, and minimal intermuscular bones, offering excellent processing adaptability. It is extensively processed into frozen fillets, surimi, and other high-value ready-to-eat products, thereby enhancing profitability and stabilizing the supply chain. Surimi is an odorless, white paste consisting of stabilized myofibrillar proteins, produced through mechanical deboning, mincing, and repeated cold-water rinsing to eliminate lipids and water-soluble components [2]. Typically, the rinsing process entails 2–3 cycles of flesh extraction and washing. Earlier research has primarily examined how rinsing parameters, such as the number of rinses, medium used, and duration, affect the gelation behavior and physicochemical properties of tilapia surimi [3,4,5,6]. These washing steps help remove endogenous enzymes, lipids, pigments, odorous compounds, and sarcoplasmic proteins, thereby reducing undesirable odors, improving product whiteness, and concentrating myofibrillar proteins to enhance gel strength [7,8]. However, repeated rinsing also leads to considerable loss of nutritious components such as proteins, fats, amino acids, and fatty acids, along with flavor and aroma compounds that contribute to the fish’s natural taste [9]. Moreover, the wastewater produced contains high levels of proteins, fats, and metal ions, raising both wastewater treatment costs and environmental impacts [10]. In light of these drawbacks associated with rinsed surimi, the development of eco-friendly green technologies for the surimi product has garnered growing interest.

Recently, unrinsed surimi has attracted more attention because the absence of a rinsing step in unrinsed surimi results in the retention of higher levels of sarcoplasmic proteins, metal ions, heme pigments, lipids, endogenous enzymes, and odor-causing compounds [11]. It also supports greater environmental sustainability by eliminating the rinsing stage. However, these remaining components can trigger protein degradation and various oxidative reactions [12]. Moreover, excessive sarcoplasmic proteins dilute the proportion of myofibrillar proteins, which compromises surimi whiteness and gel strength, ultimately leading to quality deterioration [13]. The quality of surimi-based products, especially their gelation characteristics, is influenced by various intrinsic factors (e.g., fish species, fishing season, sexual maturity, and freshness), processing approaches, and additive substances [14]. Recent research on unrinsed surimi has been largely directed toward enhancing its gel performance and oxidative stability by incorporating various natural compounds, such as proteins, polysaccharides, and antioxidants [15,16,17,18,19].

Polysaccharide complexation effectively modulates gel microstructure to enhance the mechanical properties and water retention of protein-based systems [20,21]. Various anionic (e.g., xanthan gum, alginate) and neutral (e.g., β-glucan, inulin) polysaccharides have been applied to unrinsed surimi, directly governing texture, rheology, and water holding capacity [13,15,22,23]. Above the protein isoelectric point, electrostatic repulsion and thermodynamic incompatibility favor microscale segregation into protein- and polysaccharide-rich domains [24]. This excludes proteins from the polysaccharide phase, increasing local protein concentration to promote stronger network formation [25]. Nevertheless, these approaches typically require high polysaccharide concentrations (>1.0 wt%) to achieve significant gel strengthening. Such high dosages pose economic constraints and may adversely affect sensory attributes and processability due to excessive macromolecular introduction. This highlights a critical need for alternative hydrocolloids that can deliver robust reinforcement at lower usage levels without compromising structural homogeneity.

Κ-carrageenan (κ-CG) is a sulfated linear polysaccharide derived from red algae, with a backbone composed of alternating β-(1 → 3)-D-galactose and α-(1 → 4)-3,6-anhydro-D-galactose units. It possesses a unique two-stage thermoreversible gelation mechanism, undergoing a conformational transition from random coils to helices upon cooling and subsequently assembling into a three-dimensional network via hydrogen bonding and helix aggregation [26]. The presence of cations such as K^+^ or Na^+^ further promotes helix aggregation and stabilizes the polysaccharide gel network through electrostatic shielding [27,28]. Distinct from most polysaccharides that act solely as fillers, κ-CG can form an independent gel network during the cooling phase of surimi processing, which raises the possibility of constructing a synergistic protein–polysaccharide double-network system [29,30]. However, the regulatory effects of κ-CG on unrinsed tilapia surimi gels have not been systematically elucidated.

Accordingly, κ-CG appears likely to reinforce the protein network via local microscopic phase separation and physical filling, and its cooling-induced conformational shift may contribute to supplementary structural support of the gel matrix. The present work was conducted to examine how κ-CG affects the mechanical, structural, textural, water-related, and rheological attributes of unrinsed surimi gels. Relevant physicochemical properties were also characterized in detail. The overall objective is to offer technical strategies and fundamental theoretical support for enhancing the gelling ability of unrinsed surimi, so as to advance the development of green manufacturing for surimi products using this raw material.

## 2. Results and Discussion

### 2.1. Rheological Properties Characterization

#### 2.1.1. Apparent Viscosity

**Figure 1:**
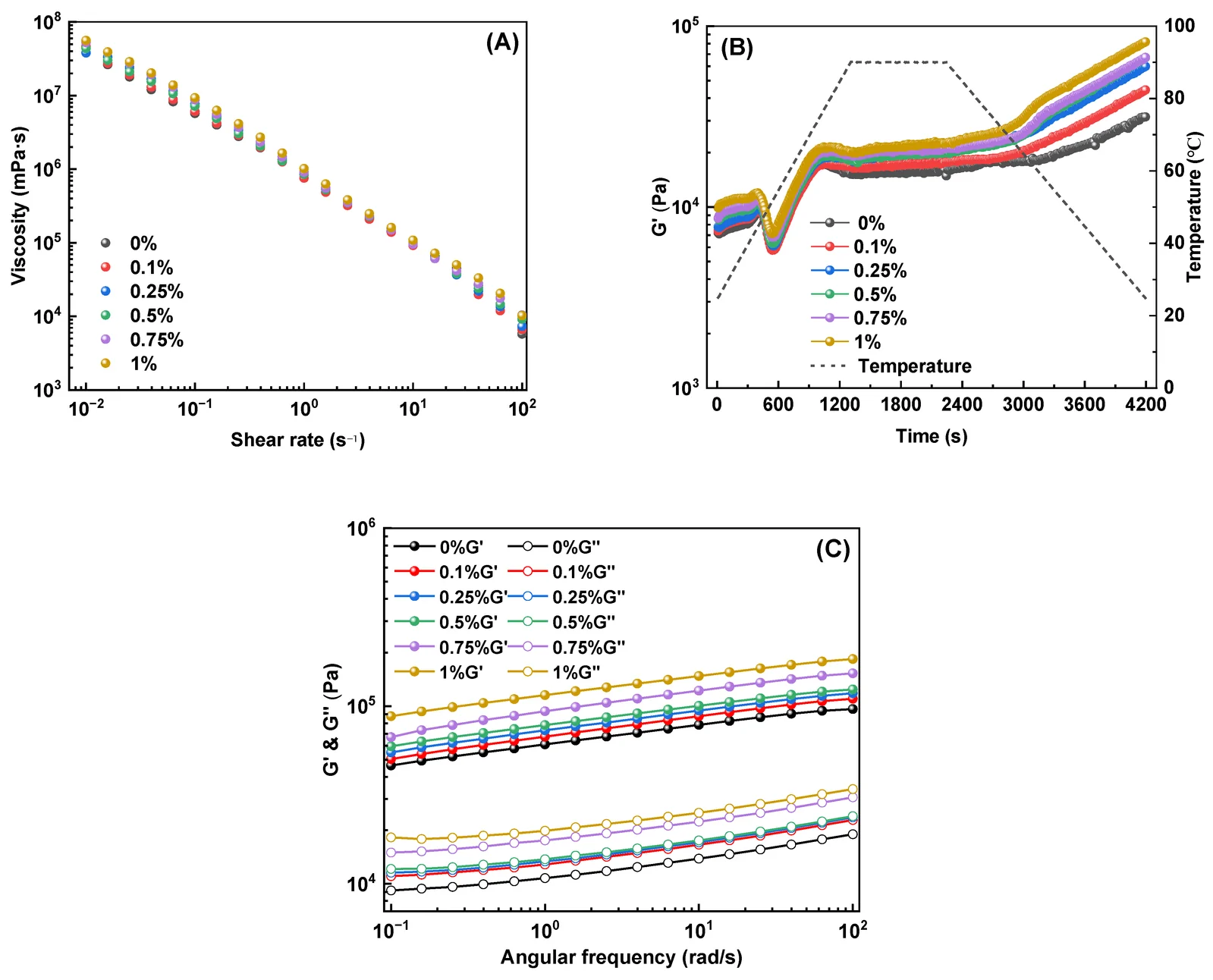
The rheological properties of tilapia surimi sols/gels with different κ-CG contents: (**A**) the viscosity of surimi sols, (**B**) the storage modulus (G′) under temperature sweep, (**C**) the storage modulus (G′) and loss modulus (G″) of surimi gels under frequency sweep.

Rheological measurements provide fundamental insights into the dynamic gelation process and network structure evolution of surimi systems, which directly determine their processability and final textural properties. As shown in Figure 1A, the apparent viscosity of unrinsed surimi sols increased gradually with increasing κ-CG concentration at 25 °C, indicating that κ-CG effectively enhanced the viscosity of the sol system even before thermal gelation. This viscosity enhancement is likely attributable to the synergistic interplay between the exceptional water-binding capacity of κ-CG and potential electrostatic interactions between its densely sulfated half-ester groups and the positively charged domains (e.g., lysine, arginine residues) of myofibrillar proteins at pH ~7.0 [14,24,31,32]. This observation is consistent with the findings of Liu et al., who reported that the addition of 0.5% κ-CG increased the viscosity of unrinsed sturgeon surimi [22].

Furthermore, all the formulations demonstrated marked shear-thinning, with apparent viscosity dropping sharply over the 0.1 to 100 s^−1^ shear rate range. This shear-thinning phenomenon is a characteristic feature of entangled biopolymer systems and arises from shear-induced rearrangement of molecular structures [33]. At low shear rates, the transient network formed by myofibrillar protein aggregates and κ-CG chains remains intact, resulting in high flow resistance. As the shear rate increases, intermolecular entanglements are disrupted, and both protein and polysaccharide molecules align along the direction of shear flow, leading to a significant reduction in viscosity. This behavior is consistent with previous reports on surimi systems modified with other polysaccharides, including konjac glucomannan [34], trehalose and fructooligosaccharides [35], and Hsian-tsao polysaccharide [36].

#### 2.1.2. Thermal Gelation Profiles

In this study, the two-step heating protocol (40 °C for 30 min, followed by 90 °C for 30 min) was used to prepare the surimi gels, which is a well-established setting-cooking procedure widely applied in surimi manufacturing [14]. The 40 °C stage is intended to promote the setting mechanism, allowing myosin heavy chains to form initial cross-links and a preliminary network structure without excessive denaturation. The subsequent 90 °C stage induces complete protein denaturation, aggregation, and the formation of a stable, three-dimensional gel matrix.

As illustrated in Figure 1B, dynamic temperature sweep tests were performed to characterize the thermal gelation behavior of surimi fortified with graded concentrations of κ-CG. The temperature protocol comprised three sequential phases: linear heating from 25 to 90 °C, isothermal holding at 90 °C, and linear cooling back to 25 °C. All the samples exhibited a conserved rheological response across these phases, with κ-CG concentration exerting a pronounced, concentration-dependent modulation of gelation kinetics and final elastic modulus.

Within the heating phase, G′ underwent three distinct transitional stages. From 25 °C to 45 °C, a slight increase in G′ was observed in all the samples, attributed to the weak preliminary network formation with minimal intermolecular association [37]. A transient reduction in G′ was observed from 45 °C to 52 °C, corresponding to the characteristic modori phase during surimi gelation [38]. This thermally induced softening is commonly attributed to the activation of endogenous serine proteases, which catalyze the hydrolysis of myosin heavy chains and disrupt nascent cross-links within the pre-gel network [39,40]. Subsequently, G′ increased exponentially in the second stage (52–80 °C), signaling the irreversible denaturation of myosin and the formation of a three-dimensional gel network. This process exposes buried hydrophobic amino acid residues and reactive sulfhydryl groups, which mediate intermolecular cross-linking via cooperative hydrophobic interactions, hydrogen bonding, and disulfide bond formation [38]. These cross-links collectively drive the assembly of a continuous three-dimensional protein network, resulting in the dramatic increase in elastic modulus. By the end of the heating phase and isothermal holding at 90 °C, G′ gradually plateaued for all the samples, signifying the completion of the majority of covalent cross-linking reactions and stabilization of the primary protein network [15]. During the final cooling phase (90–25 °C), G′ increased continuously and significantly for all the samples, with the magnitude of this increase being strongly positively correlated with κ-CG concentration (Figure 1B). Specifically, the control sample showed a moderate 2.1-fold increase in G′ from ~1.5 × 10^4^ Pa at 90 °C to ~3.2 × 10^4^ Pa at 25 °C, while the 1.0% κ-CG sample exhibited a much more substantial 3.6-fold increase from ~2.3 × 10^4^ Pa to ~8.2 × 10^4^ Pa.

This differential enhancement arises from two complementary mechanisms. First, reduced thermal motion at lower temperatures stabilizes the non-covalent intermolecular interactions (hydrogen bonds and hydrophobic forces) within the pre-formed irreversible protein network [41]. Second and predominantly, κ-CG chains undergo a reversible conformational transition from disordered random coils to ordered double helices as the temperature drops below their ion-dependent transition temperature (~40–60 °C) [28]. In the presence of Na^+^, κ-CG double helices may aggregate into stable junction zones, a process that may contribute to extra gel reinforcement [27]. The concentration-dependent rise in G′ during cooling is consistent with the possibility of interpenetrating polysaccharide domains coexisting with the protein matrix [42]. Finally, the G′ values of all the samples remained substantially higher than their initial pre-heating values, confirming the irreversible nature of myofibrillar protein denaturation and gelation.

#### 2.1.3. Frequency Sweep

Frequency sweep tests (Figure 1C) confirmed the solid-like viscoelastic behavior of all the surimi gels, as the storage modulus (G′) remained higher than the loss modulus (G″) across the tested frequency range (0.1–100 rad/s). Both moduli exhibited a slight frequency dependency, increasing with angular frequency, which is characteristic of elastic networks as commonly observed in surimi systems modified with polysaccharides [43]. The addition of κ-CG significantly enhanced both G′ and G″ in a concentration-dependent manner. At 1 rad/s, for example, the control sample showed a G′ of approximately 6.1 × 10^4^ Pa, while the 1.0% κ-CG sample exhibited a G′ of 1.2 × 10^5^ Pa, representing a more than 1.9-fold increase. κ-CG’s favorable gel-modulating performance may stem from its sulfated polysaccharide structure, which undergoes thermoreversible conformational transitions in cation-rich environments; such structural shifts are suggested to offer supplementary elastic support within the protein matrix [42,43].

### 2.2. Gel Strength and Textural Property

**Figure 2:**
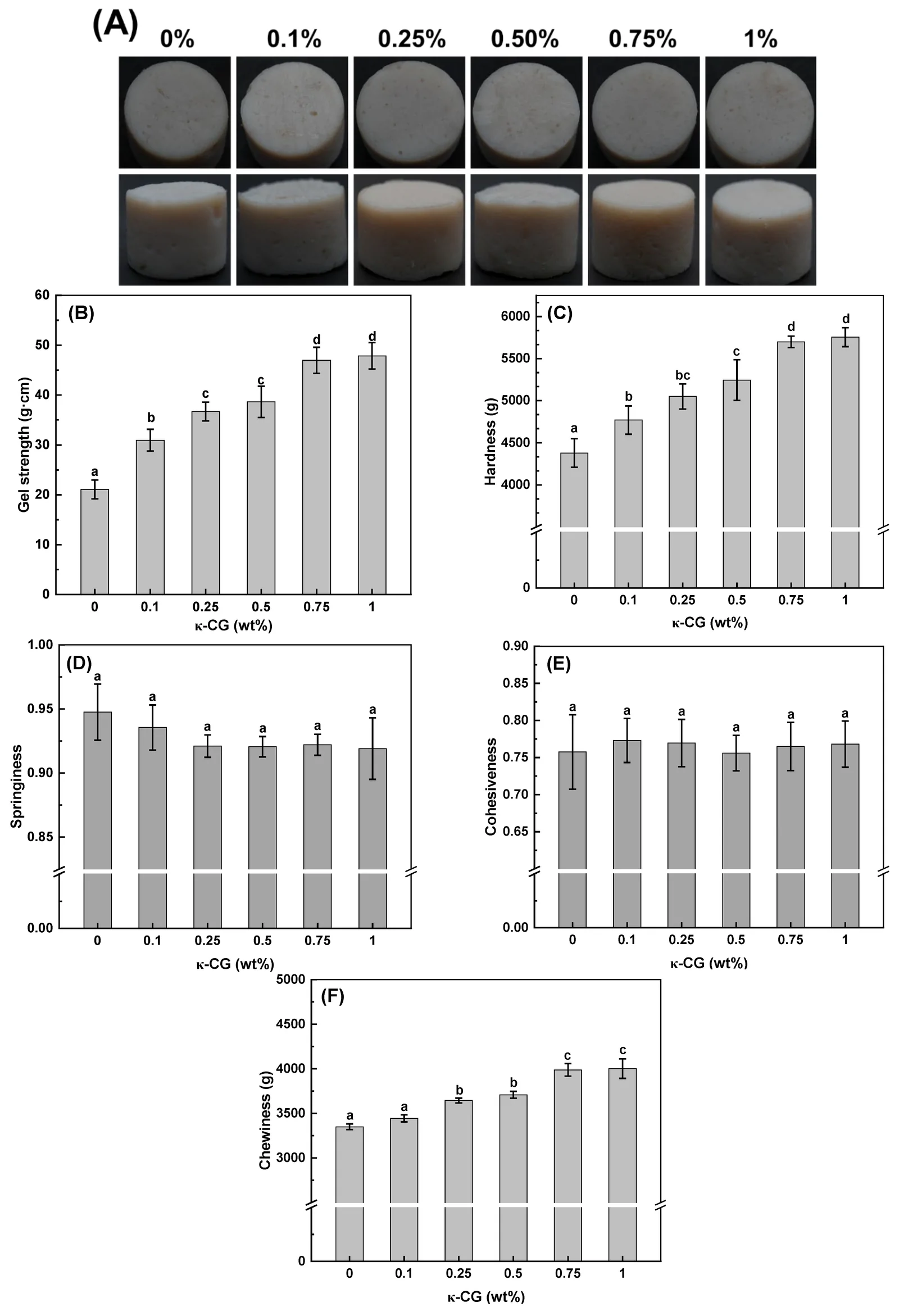
Appearance (**A**), gel strength (**B**), hardness (**C**), springiness (**D**), cohesiveness (**E**), and chewiness (**F**) of unrinsed tilapia surimi gels with different κ-CG contents. Values are mean ± standard deviation (*n* = 3); different letters indicate the significance (*p* < 0.05) among different samples.

Figure 2A presents the cross-sectional and side views of tilapia surimi gels prepared with κ-CG concentrations ranging from 0% to 1.0%. All the gels maintained intact cylindrical shapes without visible collapse or deformation throughout the entire concentration range. Notably, all the samples exhibited uniform texture with evenly distributed small pores, and no obvious macroscopic phase separation, cracks, or structural inhomogeneities were detected even at the highest κ-CG concentration.

As illustrated in Figure 2B, gel strength exhibited a monotonic increase with rising κ-CG concentration, escalating from 21.1 g·cm (0% κ-CG) to 47.8 g·cm (1.0% κ-CG). This trend is consistent with previous observations regarding κ-CG’s enhancement of gel strength in silver carp surimi [44]. However, this trend contrasts with the typical trajectory reported for many hydrocolloids, where gel strength peaks at a critical polysaccharide concentration before declining markedly. This inverted-U-shaped dose–response has been widely documented, including for *Ulva intestinalis* polysaccharide in silver carp surimi [45], β-glucan in unrinsed silver carp surimi [15], carboxymethyl cellulose in *Nemipterus virgatus* surimi [13], and tapioca starch in threadfin bream surimi [46]. The linear increase observed here indicates that κ-CG integrates uniformly into the tilapia surimi protein network across the entire 0–1.0% concentration range without inducing macroscopic phase separation. In parallel, Figure 2C reveals a consistent rise in hardness with increasing κ-CG content, from 4379 g (0% κ-CG) to 5754 g (1.0% κ-CG). This enhancement in hardness corroborates the gel strength data (Figure 2B), as both parameters reflect the stiffness of the gel network, largely governed by the density of intermolecular interactions.

Contrastingly, the textural attributes of springiness (Figure 2D) and cohesiveness (Figure 2E) remain relatively stable across the tested κ-CG concentrations (0–1.0%). Springiness, defined as the gel’s ability to recover its original height after compression, shows minor fluctuations (0.92–0.95) with a slight decrease as κ-CG concentration increased, indicating that κ-CG does not significantly alter the elastic recovery of the protein network. Cohesiveness, which reflects the energy required to deform the gel before rupture, also exhibited negligible variation (~0.75–0.77), suggesting that the internal structural integrity was preserved even with increasing κ-CG content. Chewiness is correlated with hardness, showing the increases from 3350 g to 4100 g as the κ-CG concentration increased up to 1.0% (Figure 2F). These observations collectively suggest that κ-CG primarily reinforces the tilapia surimi gel by enhancing its resistance to fracture (gel strength) (Figure 2B), compressive stiffness (hardness) (Figure 2C), and chewiness (Figure 2F), while exerting minimal influence on the gel’s elastic recovery (springiness) (Figure 2D) or internal cohesion (cohesiveness) (Figure 2E).

The differential effects of κ-CG on textural parameters can be rationalized by considering the distinct physical origins of each TPA index. Hardness and gel strength are closely associated with the density and continuity of the three-dimensional gel network, which are effectively enhanced by κ-CG through local microscopic phase separation-induced protein concentration, strengthened hydrophobic interactions and disulfide bonds, and the physical filling of voids within the matrix [44]. In contrast, springiness reflects the elastic recovery capacity of the protein backbone itself rather than the absolute network density. Therefore, the incorporation of κ-CG, which acts primarily as a filler, does not necessarily modify the intrinsic spring-back behavior of the myofibrillar protein network [23]. Cohesiveness represents the internal structural integrity of the gel, which remained intact within the tested concentration range. The increase in chewiness was essentially a secondary consequence of the remarkable improvement in hardness, given that chewiness is a derived parameter calculated as hardness × springiness × cohesiveness. Similar phenomena have been reported in other surimi–polysaccharide systems, where κ-CG or other hydrocolloids enhanced hardness and gel strength without markedly altering springiness or cohesiveness [15,23,41,47]. Thus, the selective improvement of specific textural attributes further confirms that κ-CG reinforces the surimi gel matrix mainly by densifying the network structure rather than by fundamentally altering the elastic nature of the protein network.

### 2.3. The Water Retention Analysis

#### 2.3.1. Water Holding Capacity (WHC) Analysis

**Figure 3:**
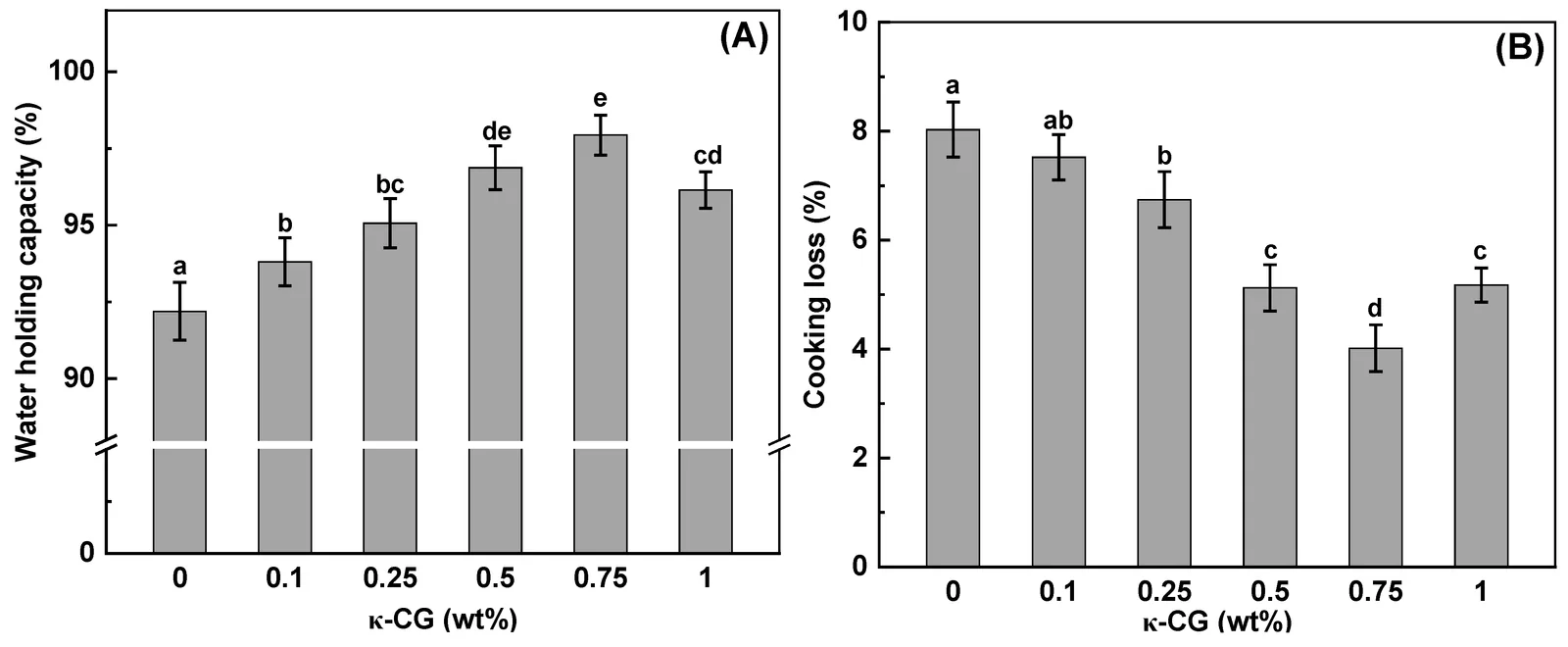
Effect of κ-CG content on water holding capacity (**A**) and cooking loss (**B**) of unrinsed tilapia surimi gels. Values are mean ± standard deviation (*n* = 3); different letters denote statistical significance (*p* < 0.05) among various samples.

The effects of κ-CG addition on the WHC and cooking loss of tilapia surimi gels are presented in Figure 3A and Figure 3B, respectively. The sample without κ-CG exhibited a WHC of 92.2% (Figure 3A) and a cooking loss of 8.0% (Figure 3B). With the increase in κ-CG concentration from 0.1% to 0.75%, the WHC of surimi gels gradually increased and reached a maximum value of 97.9% (Figure 3A). Further increasing the κ-CG concentration slightly decreased the WHC of surimi gels. Correspondingly, the cooking loss of surimi gels showed a continuous decreasing trend with increasing κ-CG concentration up to 0.75% (Figure 3B). The cooking loss decreased from 8.0% (0% κ-CG) to 4.0% at 0.75% κ-CG, and then slightly increased to 5.2% at 1.0% κ-CG. The improvement in WHC and reduction in cooking loss by κ-CG addition are consistent with previous studies on the effects of polysaccharides on surimi gel properties. Xie et al. reported that the addition of 1.0% yeast or oat β-glucan significantly increased the WHC of unrinsed silver carp surimi gels, which was attributed to the ability of β-glucan to bind large amounts of water through hydrogen bonds formed by its hydroxyl groups [15].

#### 2.3.2. Low-Field Nuclear Magnetic Resonance (LF-NMR) Analysis

**Figure 4:**
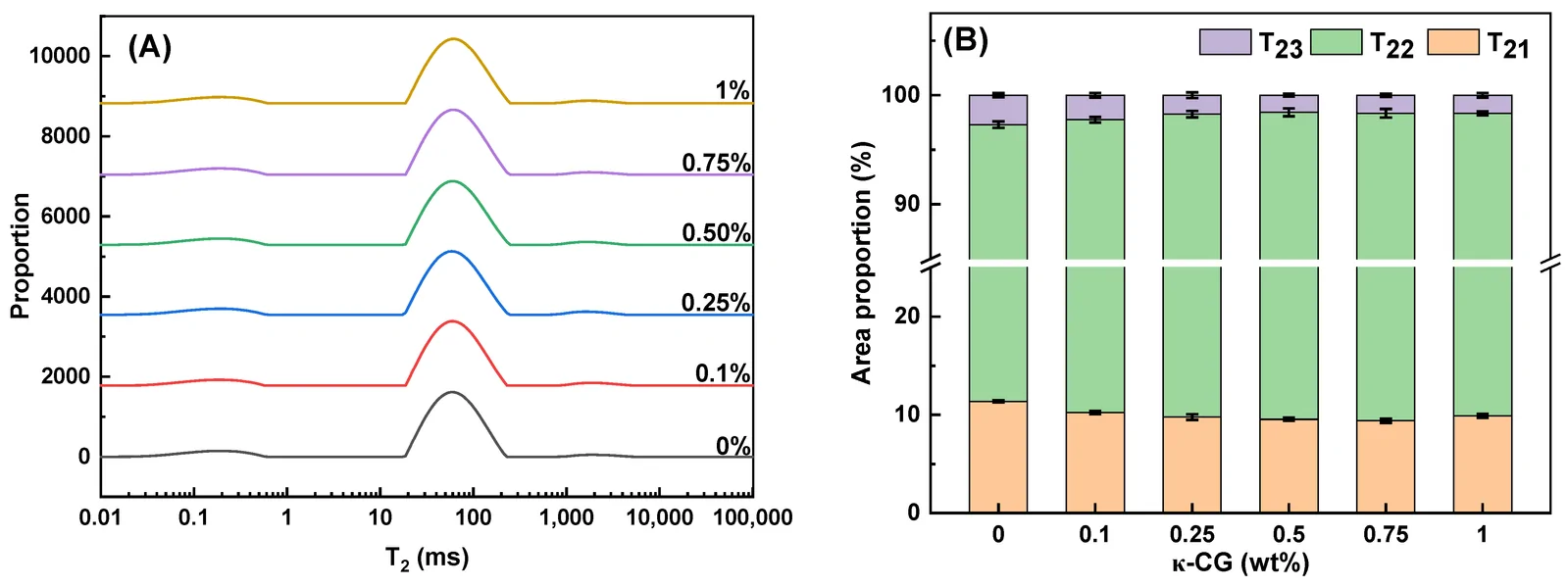
Effect of κ-CG content on the spin-spin relaxation times (T_2_) and of unrinsed tilapia surimi gels (**A**). Relative area percentages of T_21_, T_22_, and T_23_ peaks (**B**). Values are mean ± standard deviation (*n* = 3).

The LF-NMR technique was employed to investigate the water mobility and distribution in surimi gels with different κ-CG concentrations. As shown in Figure 4A, three distinct relaxation peaks were observed in all the samples, corresponding to three types of water with different binding states: T_21_ (0.1–10 ms, bound water tightly associated with macromolecules), T_22_ (10–1000 ms, immobilized water trapped in the gel network), and T_23_ (>1000 ms, free water located outside the myofibrillar lattice) [48]. The relative proportions of these three water fractions are quantified in Figure 4B. Immobilized water (T_22_) was the dominant water form in all the surimi gels, accounting for approximately 85.9–88.9% of the total water. Bound water (T_21_) accounted for about 9.4–11.4%, while free water (T_23_) only accounted for 1.6–2.7%. The addition of κ-CG slightly decreased the peak area of T_21_, which is consistent with a previous report for silver carp fish balls formulated with red seaweed polysaccharides [44]. With increasing κ-CG concentration, the proportion of T_22_ increased from 85.9% (0% κ-CG) to 88.9% (0.75% κ-CG), and the proportion of T_23_ decreased from 2.7% (0% κ-CG) to 1.7% (0.75% κ-CG). The further increase in the κ-CG concentration to 1.0% resulted in a slight increase in T_22_ to 88.4%. These results indicate that κ-CG promotes the conversion of free water to immobile water, which is trapped within the gel network. This is consistent with the WHC results (Figure 3A), as immobile water is more difficult to remove than free water. Similar findings were reported by Si et al., who found that ultrasound combined with citrus pectin increased the immobile water content in low-salt silver carp surimi gels [49].

The underlying mechanisms by which κ-CG improves the water retention properties of surimi gels can be attributed to three main aspects. First, κ-CG exhibits excellent water absorption and swelling properties. During the heating process of surimi gel formation, κ-CG molecules absorb water and swell, filling the interstitial spaces in the three-dimensional network formed by myofibrillar proteins. This physical filling effect effectively blocks the channels through which water would otherwise escape during cooking, thus reducing cooking loss (Figure 3B) [50]. Second, as an anionic polysaccharide, κ-CG contains abundant sulfate ester and hydroxyl groups. These polar moieties form extensive hydrogen bonds with amino/carboxyl groups of myofibrillar proteins and water molecules, strengthening protein-water associations and converting loosely bound free water into tightly immobilized water [44,49,51]. Third, κ-CG can promote the unfolding and cross-linking of myofibrillar proteins, leading to the formation of a more compact and uniform gel network with smaller pore sizes. A denser gel network has a greater capacity to retain water, as demonstrated by the reduced average pore size observed in surimi gels with appropriate polysaccharide addition [42].

### 2.4. Gel Formation Mechanism

#### 2.4.1. FT-IR

**Figure 5:**
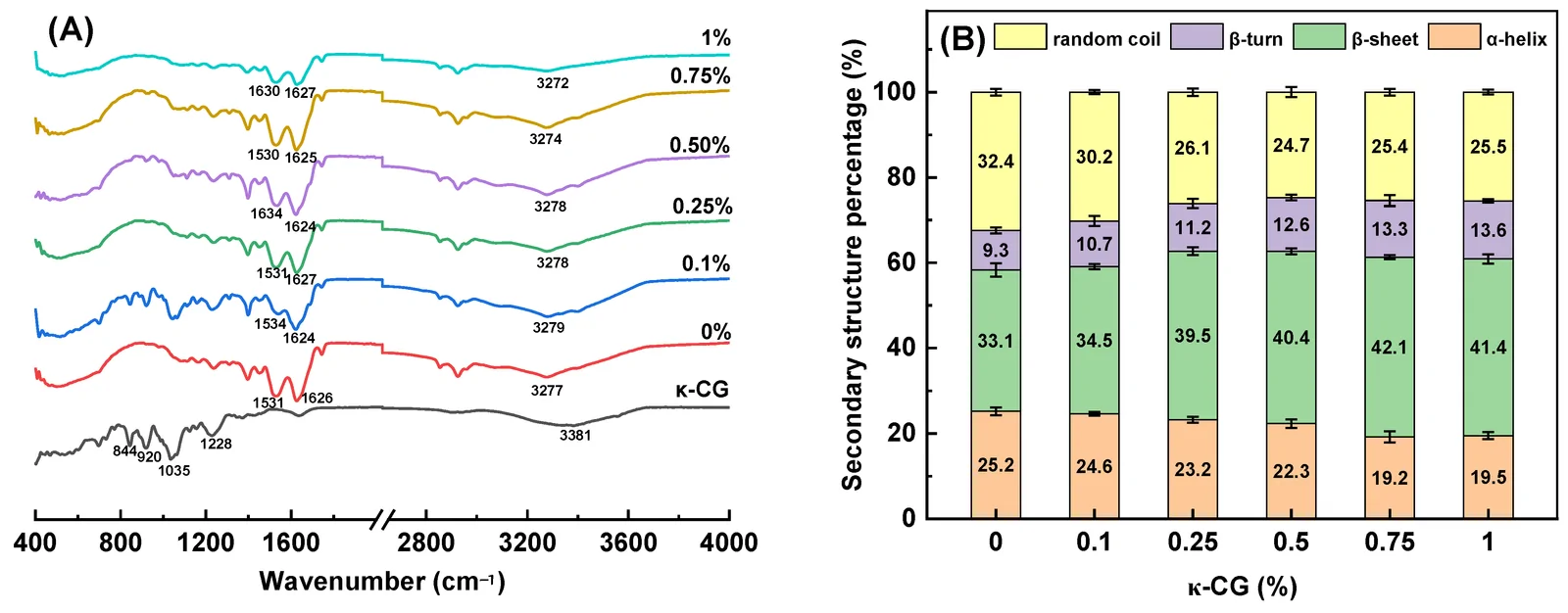
Fourier transform infrared (FTIR) spectra (**A**) and secondary structure (**B**) of κ-CG and unrinsed tilapia surimi gels with various concentrations of κ-CG.

FT-IR was utilized to investigate the molecular interactions between κ-CG and myofibrillar proteins. In Figure 5A, the pure κ-CG spectrum exhibits characteristic absorption bands at 928 cm^−1^ (3,6-anhydrogalactose C-O-C stretching), 1040 cm^−1^ (glycosidic bond), and 1228 cm^−1^ (ester sulfate S=O stretching), which are consistent with previously reported spectral features of κ-CG [52]. In the surimi gel spectra, these κ-CG-specific bands become less prominent, confirming the successful incorporation of κ-CG into the gel matrix.

The broad absorption band centered at approximately 3280 cm^−1^ was observed in the surimi gels in the absence and presence of κ-CG (Figure 5A), attributed to the O-H stretching vibrations of hydroxyl groups involved in hydrogen-bonding networks [53]. This result indicates that intermolecular and intramolecular hydrogen bond forces are present in the surimi gels [49]. Notably, as κ-CG content increased, this O–H band shifted progressively toward lower wavenumbers (from 3277 cm^−1^ for the control to 3272 cm^−1^ for 1% κ-CG), accompanied by a significant increase in peak intensity. The red shift in the O–H stretching band is a well-documented spectroscopic indicator of enhanced intermolecular hydrogen bonding: stronger hydrogen bonds reduce the force constant of O–H covalent bonds, leading to a decrease in vibrational frequency [54,55]. This result indicated that κ-CG formed additional intermolecular hydrogen bonds with myofibrillar proteins during gelation, which acted as cross-linking points to stabilize the three-dimensional gel network.

The amide I band (1600–1700 cm^−1^), originating primarily from C=O stretching vibrations of peptide bonds, is the most sensitive spectral region for monitoring changes in protein secondary structure (Figure 5A). To quantify these changes, the amide I region was subjected to second-derivative calculation followed by Gaussian curve fitting (Figure 5B). As shown in Figure 5B, the control sample (0% κ-CG) displayed a typical α-helix-dominant structure, with the α-helix content calculated at 25.2% and β-sheet content at 33.1%. Upon the addition of κ-CG, significant alterations in the secondary structure composition were observed. The α-helix content progressively decreased from 25.2% (control) to 19.5% (1% κ-CG), while the β-sheet content substantially increased from 33.1% to a maximum of 42.1% at 0.75% κ-CG before slightly receding to 41.4% (Figure 5B). This quantifiable shift indicates that the incorporation of κ-CG facilitates the transformation of the protein backbone from α-helices to β-sheets during the thermal gelation process. A similar phenomenon could be observed in pork myofibrillar proteins with κ-CG [31], oyster protein with κ-CG [56], and golden pompano surimi gel with κ-CG [57]. The increased β-sheet content suggests the formation of a more ordered, compact, and stable protein network, which is a hallmark of strong heat-induced gelation [47]. Conversely, the proportions of β-turns and random coils showed minor fluctuations across the different concentrations, suggesting that the primary structural rearrangement is concentrated on the helix-to-sheet transition.

#### 2.4.2. Intermolecular Interaction Contributions

**Figure 6:**
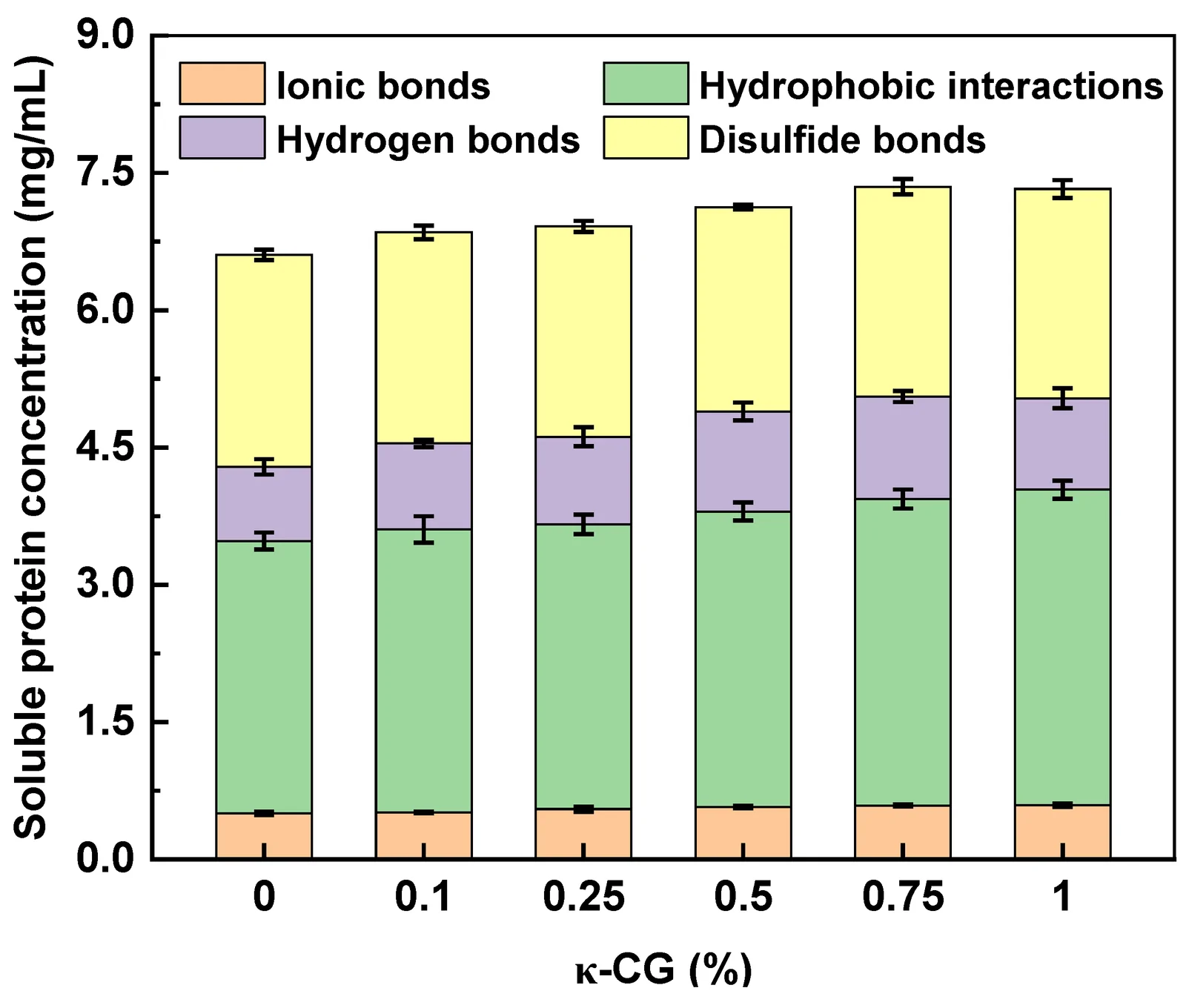
Chemical forces of unrinsed tilapia surimi gels with various concentrations of κ-CG. Values are mean ± standard deviation (*n* = 3).

Figure 6 shows the relative solubility indices associated with ionic bonds, hydrophobic interactions, hydrogen bonds, and disulfide bonds in surimi gels under different treatments, as derived from solvent extraction assays. Based on this operationally defined assay, hydrophobic interactions and disulfide bonds appeared to be the major contributors driving gel network formation (Figure 6). Thermal treatment induced protein denaturation and exposed hydrophobic patches on the myosin head, thereby facilitating protein cross-linking and aggregation [58]. Additionally, heating accelerates sulfhydryl oxidation, resulting in the formation of disulfide bonds. Consistent with previous findings [49,59], hydrophobic interactions and disulfide bonds are essential for the development of surimi gel networks. Notably, κ-CG addition effectively augmented hydrophobic interactions (Figure 6) and consequently boosted surimi gel strength (Figure 2B & C). This observation could be explained by the fact that κ-CG functions as a filler in the gel matrix, facilitating local protein aggregation and thereby increasing the local concentration of myofibrillar proteins, which indirectly promotes hydrophobic interactions [60].

As indicated by the solubility assay, hydrogen bonds and ionic bonds did not appear to be the dominant stabilizing forces for the surimi gel networks (Figure 6), which aligns with the findings from silver carp surimi gels with κ-CG [47,61], scallop surimi gels containing κ-CG [50], and Antarctic krill surimi gels incorporating sodium alginate [62]. With increasing κ-CG concentration, the hydrogen bond index first rose slightly and then declined at the highest κ-CG concentration. Low concentrations of polysaccharides could associate with free water via hydrogen bond formation. Nevertheless, excessive hydrocolloid addition inhibited complete protein unfolding and subsequent hydrogen bonding between proteins and water molecules, resulting in decreased WHC [63]. This observation was in good agreement with the WHC data presented in Figure 3A. Additionally, a slight increase in ionic bonding was observed with rising κ-CG levels (Figure 6). This is likely due to the neutralization of surface-exposed cationic groups on the protein surface by the negatively charged κ-CG molecules at neutral pH [32,64].

### 2.5. Microstructure of Surimi Gels

**Figure 7:**
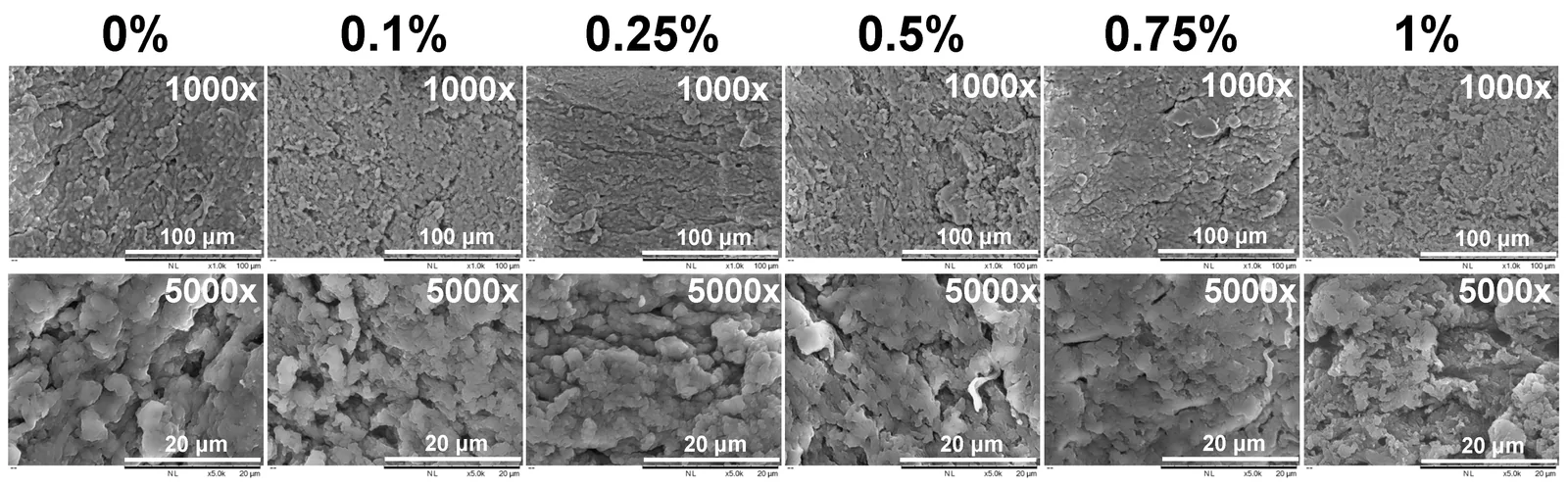
Microstructure of unrinsed tilapia surimi gels with various concentrations of κ-CG.

Figure 7 presents SEM micrographs detailing the microstructural evolution of tilapia surimi gels as a function of κ-CG concentration, and the corresponding quantitative structural parameters are summarized in Table 1. The surimi gel without κ-CG (control group) exhibited the highest pore number (4862 ± 608) and apparent porosity (9.277 ± 1.121%), along with the lowest fractal dimension (*D_f_* = 1.842 ± 0.009) and a relatively high lacunarity (0.181 ± 0.002). These quantitative results confirmed the visual observation of a loose, discontinuous, and heterogeneous microstructure with widely distributed pores of varying sizes. The low *D_f_* indicated a low degree of spatial filling and poor cross-linking density of the protein network, while the high lacunarity reflected remarkable heterogeneity in pore size distribution [65,66]. Such a defective microstructural framework directly accounted for the inferior gel strength (Figure 2), poor WHC, and high cooking loss of the control sample (Figure 3).

As κ-CG dosage rose from 0% to 0.75%, key structural parameters did not follow a simple monotonic trend at intermediate concentrations, yet the comprehensive microstructural performance progressively improved and peaked at 0.75%. At 0.75% κ-CG, the apparent porosity and average pore area of the gels reached minimum values of 4.937 ± 0.341% and 0.378 ± 0.009 μm^2^, respectively. Meanwhile, *D_f_* increased to a maximum of 1.872 ± 0.006, and lacunarity decreased to a minimum of 0.177 ± 0.000, indicating that the protein network became more compact, continuous, and spatially filled, with a more uniform pore size distribution [67]. These quantitative findings corroborated the morphological observation of a finer and more ordered network structure. The improved microstructural homogeneity and compactness could be attributed to intermolecular interactions between κ-CG and myofibrillar proteins during heating. The anionic κ-CG molecules interacted with protein aggregates via hydrogen bonds and hydrophobic interactions, which inhibited excessive and uncontrolled protein aggregation and promoted the formation of a uniform cross-linked network [50]. This well-constructed microstructure with small and homogeneous pores provided more capillary binding sites for water molecules, thus enhancing WHC and reducing cooking loss [42]. In addition, the dense and continuous protein network endowed the gel with higher mechanical resistance, corresponding well to the elevated gel strength at 0.75% κ-CG (Figure 2).

Notably, further increasing κ-CG to 1.0% led to a significant increase in average pore area (0.581 ± 0.002 μm^2^) and lacunarity (0.183 ± 0.000), along with a decrease in *D_f_* (1.858 ± 0.001) and pore number (2128 ± 397). These quantitative data demonstrated that excessive κ-CG induced the formation of large, irregular cavities and disrupted the structural uniformity of the gel network, which was consistent with the fragmented and rough morphology observed in SEM images (Figure 7). This microstructural deterioration could be explained by the depletion flocculation effect: high concentrations of κ-CG induced local microscopic phase separation between the polysaccharide and protein phases, resulting in the formation of large voids and weakened network integrity [68]. The increased pore size and structural heterogeneity consequently weakened the water immobilization ability, explaining the reduced WHC and increased cooking loss (Figure 3) at 1.0% κ-CG.

## 3. Conclusions

This study systematically investigated the potential of κ-CG as a functional additive to overcome the quality defects of unrinsed Nile tilapia surimi, demonstrating that κ-CG enhances gel properties in a concentration-dependent manner primarily by promoting protein cross-linking and optimizing water distribution. While gel strength and hardness increased monotonically up to 1.0% κ-CG, the 0.75% addition achieved the highest water holding capacity (97.9%), lowest cooking loss (4.0%), and a refined microstructure characterized by minimal porosity and maximal fractal dimension. Conversely, exceeding this concentration to 1.0% led to compromised water retention, increased average pore area, elevated lacunarity, and structural deterioration likely attributable to local microscopic phase separation rather than definitive macroscopic phase separation. Physicochemical characterization suggests the enhancement is driven by the synergistic integration of κ-CG into the protein matrix, which appears to reinforce hydrophobic interactions and disulfide bonds while converting free water into immobilized water. Although rheological and microstructural data imply the formation of a composite network, the existence of a covalently bonded dual-network structure requires further verification via advanced in situ characterization techniques. From an industrial perspective, incorporating κ-CG presents a viable strategy for sustainable surimi production by eliminating water-intensive rinsing, thereby reducing wastewater discharge and preserving nutrients to lower production costs and meet clean-label demands. Future research should focus on pilot-scale validation, sensory evaluation, and exploring the cryoprotective effects during frozen storage to ensure commercial feasibility.

## 4. Materials and Methods

### 4.1. Materials

Live Nile tilapia (*Oreochromis niloticus*) with a length of 26.6 ± 3.9 cm and weight of 798.6 ± 93.4 g were purchased from the local market (Wuxi, China). κ-CG was purchased from Sigma-Aldrich Co. (St. Louis, MO, USA). All the analytical-grade chemicals were supplied by SinoPharm CNCM Ltd. (Shanghai, China). Deionized water used for sample preparation was produced with a Milli-Q water purification system (Millipore, Billerica, MA, USA).

### 4.2. Preparation of Unrinsed Surimi Sols and Gels

Unrinsed surimi sols and gels were prepared in accordance with the established method reported in reference [13] with detailed specifications as follows. Fresh tilapia was first percussively stunned and slaughtered by professional operators, then transported to the laboratory on crushed ice within 30 min at temperatures under 4 °C to preserve raw material freshness. After removing internal organs and rinsing thoroughly, dorsal white muscle was collected and homogenized for 2 min using a JR90S-G40 mincing machine (Zhejiang Supor Co., Ltd., Zhejiang, China). Sodium chloride was added at 25 g/kg of fish muscle, and the mixture was homogenized for an additional 2 min. Different levels of κ-CG (0, 0.1, 0.25, 0.5, 0.75, and 1 wt%, based on the total mass of fish muscle) were blended into the minced fish, with a further 5 min of homogenization. The concentration range of κ-CG was determined primarily based on our previous work on pea protein-κ-CG double-network gel systems, which demonstrated that κ-CG at concentrations below 1.0 wt% effectively enhances the mechanical strength of protein gel matrices [42]. Ice-cold deionized water was used to regulate the final moisture content of surimi sols to 80 g/100 g, and all the operations were conducted below 10 °C. The prepared surimi sols were transferred into plastic tubes with an inner diameter of 25 mm, which were then sealed tightly to avoid moisture loss during heating. A two-stage heat-induced gelation procedure was applied, consisting of incubation at 40 °C for 30 min followed by heating at 90 °C for 30 min [15]. After thermal treatment, the gels were immediately quenched in ice water for 15 min and then stored at 4 °C overnight before subsequent analyses.

### 4.3. Rheological Property Measurement

The rheological property measurements were done with a Rotational Rheometer (MCR92, Anton Paar GmbH, Graz, Austria) equipped with a parallel plate geometry (25 mm in diameter) to investigate the apparent viscosity (*η*) of surimi sols, the thermal gelation process from surimi sol to gel transition, and viscoelastic properties of surimi gels according to an established protocol [15].

To determine the linear viscoelastic region (LVR), a strain amplitude sweep was first performed at 25 °C with a fixed gap of 1 mm and a constant angular frequency of 1.0 Hz. The strain range was set from 0.01% to 100% to identify the limit of the LVR. All the subsequent oscillatory tests were conducted within this confirmed LVR to ensure data validity.

Apparent viscosity of surimi sols was determined at 25 °C under a fixed gap of 1 mm, with shear rates varying between 0.1 and 100 s^−1^.

Dynamic temperature sweep tests were carried out using a parallel-plate fixture with a diameter of 25 mm. Surimi sol was loaded onto the bottom plate, and the gap between the two plates was adjusted to 1 mm. Silicone oil was coated on the sample periphery to avoid water evaporation throughout the heating and cooling process. The sample temperature was controlled by a Peltier device. Measurements of storage modulus (G′) and loss modulus (G″) were performed at 10 rad/s and 0.01% strain (linear viscoelastic range). The samples were heated from 25 °C to 90 °C at 2 °C/min and maintained for 15 min, then cooled down to 25 °C at a rate of 4 °C/min.

Frequency sweep tests for surimi gels were implemented at 25 °C with 0.25% strain (linear viscoelastic region). Angular frequency was scanned over 0.628–100 rad/s, and the variations in G′ and G″ were recorded to analyze the viscoelastic properties of different gel systems.

### 4.4. Gel Strength Measurement

A TA-XT-Plus texture analyzer (Stable Micro Systems Ltd., Godalming, UK) was employed to determine the gel strength of surimi gels [41]. The P/25N cylindrical probe (2.5 mm diameter) was adopted for the test, with operating parameters set at 2.0 mm/s (pre-test speed), 1.0 mm/s (test speed), 3.0 mm/s (post-test speed), 5 mm (compression distance), and 10 g (trigger force). Each surimi gel was prepared into cylindrical samples measuring 20 mm in length and 25 mm in diameter. A total of ten parallel tests were conducted, and gel strength values were calculated via Equation (1).

Gel strength (g × cm) = breaking force (g) × deformation (cm)(1)

### 4.5. Texture Profile Analysis (TPA)

The TPA of surimi gels was carried out based on the method described in our earlier work [42]. Cylindrical surimi samples (20 mm height, 20 mm diameter) were loaded onto the TA-XT-Plus texture analyzer fitted with a 36 mm P/36R cylindrical probe. The instrument was operated at a trigger force of 10 g, while pre-test, test, and post-test speeds were set to 5.0, 3.0, and 3.0 mm/s, respectively, under a compression strain of 50%. Textural parameters were derived from the resultant force-time curves using Exponent software (Version 8). Four key textural indices were acquired with five parallel measurements for each group. Hardness is expressed in grams (g) and corresponds to the peak force of the first compression cycle. Springiness is dimensionless and defined as the ratio of the height of the second compression to that of the first compression. Cohesiveness is dimensionless and calculated as the ratio of the positive force area during the second compression to that during the first compression. Chewiness is expressed in grams (g) and derived from the product of hardness, springiness, and cohesiveness. Five parallel measurements were performed for each group.

### 4.6. Water Holding Capacity (WHC)

WHC of unrinsed surimi gels was evaluated in accordance with the experimental procedure described in a previous study [23]. Gel samples were cut into 3 mm-thick slices and weighed to obtain the initial mass (*W*_1_). Each slice was wrapped in three pieces of filter paper and loaded into a centrifuge tube. The samples were centrifuged at 1677 g for 15 min at 4 °C. The post-centrifugal weight (*W*_2_) was then recorded, and WHC was calculated via Equation (2).

WHC (%) = *W*_2_/*W*_1_ × 100 (2)

### 4.7. Cooking Loss

The cooking loss of surimi gels was evaluated according to a previously published protocol with slight adjustments [37]. Gel specimens were sectioned into 3 mm-thick slices and weighed to obtain the initial mass (*G*_1_). These slices were sealed in cooking bags and subjected to a 20 min water-bath treatment at 90 °C. Once the surface water was wiped off, the post-cooking weight (*G*_2_) was documented. Equation (3) was applied to calculate the cooking loss rate.

Cooking loss (%) = (*G*_1_ − *G*_2_)/*G*_1_ × 100 (3)

### 4.8. Low-Field Nuclear Magnetic Resonance (LF-NMR)

An LF-NMR analyzer (MicroMR12-60, Niumag, Shanghai, China) was applied to determine the spin–spin relaxation time (T_2_) of tilapia surimi gels [34]. All the gel samples were processed into 25 mm cylindrical shapes and covered with polyethylene plastic before measurement. The waiting time, echo number, echo interval, and scan number were set to 5000 ms, 20,000, 0.1 ms, and 8, respectively. Finally, we calculated the inherent (T_2_) peak positions and the relative percentage of each peak component.

### 4.9. Estimation of Intermolecular Interaction Contributions

The intermolecular forces maintaining the structural integrity of surimi gels were evaluated following an established method described in the literature [59]. Aliquots (2 g) of surimi gels were individually homogenized for 1 min in 10 mL of the respective extracting solutions: S1 (0.05 mol/L NaCl), S2 (0.6 mol/L NaCl), S3 (0.6 mol/L NaCl + 1.5 mol/L urea), S4 (0.6 mol/L NaCl + 8 mol/L urea), and S5 (0.6 mol/L NaCl + 8 mol/L urea + 0.5 mol/L 2-β-mercaptoethanol). All the homogenates were kept at 4 °C for 1 h and then centrifuged at 10,000× *g* for 15 min. The supernatants were separated from the sediment after centrifugation. The concentration of solubilized protein in each group was quantified using the Kjeldahl assay, using a conversion factor of 6.25 for nitrogen to protein. The indices corresponding to four major intermolecular forces were calculated based on the following formulas:

Index of ionic bonds = S2 − S1(4)

Index of hydrogen bonds = S3 − S2(5)

Index of hydrophobic interactions = S4 − S3(6)

Index of disulfide bonds = S5 − S4(7)

### 4.10. Fourier Infrared Spectroscopy (FTIR)

Freeze-dried gel samples were ground with KBr in a 1:100 (*w*/*w*) mass ratio and homogenized to form a uniform powder. FTIR spectral data were collected using a Nicolet iS10 spectrometer (Thermo Fisher Scientific, Waltham, MA, USA). FTIR spectra were collected from 4000 to 400 cm^−1^. The spectral resolution was set to 4 cm^−1^, and each sample was scanned 32 times, while pure KBr was used for background subtraction. Fourier self-deconvolution was applied to analyze the secondary structure of samples. The amide I band (1600–1700 cm^−1^) was deconvoluted using OMNIC 9.2 software. Spectral resolution was optimized by applying a Fourier self-deconvolution algorithm with a fixed bandwidth of 24 cm^−1^ and an enhancement factor of 2.5 to resolve individual component peaks [69].

### 4.11. Scanning Electron Microscopy (SEM)

For microscopic analysis, 1 g of surimi gel specimens was fixed by soaking in 2.5% (v/v) glutaraldehyde at 4 °C over 24 h, followed by triple rinses with 0.1 M phosphate buffer (pH 7.0) [42]. The treated samples were freeze-dried and trimmed into rectangular slices (5 mm × 5 mm × 2 mm) using a razor blade. All the specimens were sputter-coated with gold using a JEOL JFC-1600 ion sputter coater (JEOL Ltd., Tokyo, Japan) under the following conditions: Au target, sputtering accelerating voltage of 1.0 kV, sputtering current of 20 mA, sputtering time of 60 s, working distance of 40 mm, and sputtering working pressure of approximately 8 Pa. The coated samples were examined with a JEOL 7500F field-emission scanning electron microscope (JEOL Ltd., Tokyo, Japan). Imaging was performed under an accelerating voltage of 15 kV, working distance 6 mm, observation chamber pressure around 1 × 10^−4^ Pa, and emission current 32,500 nA. Images were acquired at magnifications of 1000× and 5000×.

The microstructure of surimi gel was observed via SEM at 1,000× magnification. All the micrographs were quantitatively analyzed using ImageJ 2.16.0 software coupled with the FracLac 2.5 Release 1d plugin [67,70]. Briefly, SEM images were converted to 8-bit grayscale and calibrated with the embedded scale bar to convert pixel units into micrometers. Two independent binary images were generated with fixed threshold values: the median grey threshold (116 ± 8) for fractal dimension calculation, and the threshold (73 ± 2) yielding maximum pore count (73 ± 2) for pore morphological statistics [71]. The Analyze Particles function was applied to quantify pore number and average pore area. Apparent porosity was calculated as the ratio of total pore area to total image area. The standard box-counting algorithm in FracLac was used to determine the 2D fractal dimension, and the 3D fractal dimension (*D_f_*) was derived by adding 1 to the 2D value to represent the spatial filling characteristics of the three-dimensional gel network. Lacunarity, a parameter reflecting the heterogeneity of pore size and spatial distribution, was simultaneously exported from the FracLac analysis. Three biological replicates with multiple SEM fields per sample were analyzed for statistical reliability.

### 4.12. Statistical Analysis

All the experiments were performed with three independent surimi gel preparation batches as biological replicates, and all the statistical analyses were conducted on the basis of biological replicates. For gel strength measurement, 10 technical replicate samples were tested per batch. For texture profile analysis, 5 technical replicate samples were analyzed per batch. For other physicochemical assays including rheological measurement, water holding capacity, cooking loss, low-field nuclear magnetic resonance, and intermolecular force determination, each assay was performed directly across the three independent batches with one test per batch, and no additional technical replicates were included. All data are reported as mean ± standard deviation (SD). Before statistical analysis, the Shapiro–Wilk test and Levene’s test were performed to verify the normality of data distribution and homogeneity of variances, respectively. Statistical evaluation was conducted via one-way analysis of variance (ANOVA) followed by Duncan’s multiple range test for post hoc comparison using SPSS 22.0 software (IBM, Armonk, NY, USA). Differences were regarded as statistically significant at *p* < 0.05.

## Figures and Tables

**Table 1:** Pore number, average pore area, apparent porosity, lacunarity, and fractal dimension (*D_f_*) of tilapia surimi gels with different κ-CG concentrations, determined from binary SEM images.

κ-CG (%)	Pore Number	Average Pore Area (μm^2^)	Apparent Porosity (%)	Lacunarity	*D_f_*
0	4862 ± 608 ^a^	0.459 ± 0.049 ^a^	9.277 ± 1.121 ^a^	0.181 ± 0.002 ^ab^	1.842 ± 0.009 ^a^
0.1	3459 ± 167 ^b^	0.613 ± 0.067 ^b^	8.024 ± 1.255 ^ab^	0.186 ± 0.001 ^c^	1.865 ± 0.000 ^cd^
0.25	3106 ± 496 ^b^	0.731 ± 0.124 ^c^	8.325 ± 0.088 ^ab^	0.182 ± 0.000 ^ab^	1.847 ± 0.009 ^ab^
0.5	3256 ± 141 ^b^	0.575 ± 0.008 ^b^	7.040 ± 0.396 ^b^	0.180 ± 0.002 ^a^	1.859 ± 0.008 ^bc^
0.75	3469 ± 163 ^b^	0.378 ± 0.009 ^a^	4.937 ± 0.341 ^c^	0.177 ± 0.000 ^d^	1.872 ± 0.006 ^d^
1	2128 ± 397 ^c^	0.581 ± 0.002 ^b^	4.658 ± 0.879 ^c^	0.183 ± 0.000 ^b^	1.858 ± 0.001 ^b^

Different letters in the same column indicate statistically significant differences (*p* < 0.05).

## Data Availability

The raw data supporting the conclusions of this article will be made available by the authors on request.
